# Ações Transmurais Inotrópicas e Antiarrítmicas da Ranolazina em um Modelo Celular da Síndrome do QT Longo Tipo 3

**DOI:** 10.36660/abc.20190220

**Published:** 2020-05-12

**Authors:** Victor Martins Miranda, Samuel Santos Beserra, Danilo Roman Campos

**Affiliations:** 1 Universidade Federal de São Paulo Departamento de Biofísica São Paulo SP Brasil Universidade Federal de São Paulo, Departamento de Biofísica, Edifício de Ciências Biomédicas, São Paulo, SP - Brasil

**Keywords:** Arritmias, Síndrome do QT Longo do Tipo 3, ATX-II, Ranolazina, Corrente Tardia de Sódio, Contração

## Abstract

A Ranolazina (RANO), conhecida na clínica como Ranexa, é um fármaco que previne a arritmia cardíaca através da inibição da corrente de sódio tardia (I_NaT_). Um gradiente de voltagem transmural do canal Nav1.5 encontra-se na parede ventricular esquerda do coração. Assim, investigamos os efeitos da RANO em cardiomiócitos saudáveis e em modelo celular da Síndrome do QT longo tipo 3 (SQTL tipo 3). Usamos células isoladas do endocárdio (ENDO) e do epicárdio (EPI) e um *software* de medição com detecção de bordas por vídeo e microscopia de fluorescência para monitorar os transientes de cálcio. A RANO (0,1, 1, 10 e 30 uM, a 25^O^C) em uma série de frequências de estimulação teve impacto pouco significativo sobre ambos os tipos de células, mas a RANO (30uM) a 35^O^C minimizou o encurtamento dos sarcômeros em ~21% para células do endocárdio. Em seguida, para simular a SQTL tipo 3, as células do ENDO e EPI foram expostas à toxina ATX-II da anêmona do mar, que aumenta a I_NaT_. As arritmias celulares induzidas por ATX-II foram suprimidas com o uso da RANO (30 µM) a 35^O^C. Com base nesses resultados, podemos concluir que a RANO tem um impacto pouco significativo sobre o encurtamento dos sarcômeros de células saudáveis do ENDO e EPI. Além disso, ela suprime as arritmias induzidas por I_NaT_ para níveis semelhantes nas células do ENDO e EPI.

## Introdução

A arritmia nas doenças cardiovasculares é uma das principais causas de morte no mundo todo.^1^ A ação antiarrítmica da ranolazina é atribuída à diminuição do componente de inativação lenta da corrente cardíaca interna através do Nav1.5, conhecida como corrente de sódio tardia (I_NaT_).^2^ Apesar dos avanços importantes na compreensão dos mecanismos celulares subjacentes à ação da RANO, a sua ação transmural nas células musculares do coração permanece incerta. Consequentemente, neste estudo, nossa hipótese é que a RANO desempenha uma ação transmural nas células saudáveis do endocárdio (ENDO) e epicárdio (EPI) estimuladas por região, bem como nas arritmias e perturbação do cálcio induzidas pela toxina de anêmona (ATX-II),^3^ que aumenta a I_NaT_ e simula vários aspectos da Síndrome do QT longo tipo 3 (SQTL tipo 3), uma doença relacionada ao aumento da I_NaT_ nas células do coração.^2^

## Métodos

### Animais

Foram usados ratos Wistar machos (160-250 g; com 5 a 7 semanas de vida) nos experimentos. Todos os procedimentos experimentais foram realizados conforme as diretrizes institucionais, e o estudo foi aprovado pelo Comitê de revisão ética local. Os cardiomiócitos foram isolados conforme descrito anteriormente.^4^

### O encurtamento do sarcômero e o transiente de cálcio

Os experimentos foram conduzidos como descrito anteriormente pelo nosso grupo.^5^ As células foram perfundidas com RANO (Alomone, Israel) a 0,1, 1, 10, ou 30 µM a partir de uma solução stock de 10 mM. Os dados foram normalizados como a função de contração do sarcômero antes da exposição à RANO. Para acessar o efeito antiarrítmico da RANO após exposição ao ATX-II (6 nM) (Alomone, Israel), os tempos para 90% de relaxamento do sarcômero (T90R) e recaptação de cálcio (T90Ca^2^) foram registrados como índices arrítmicos. Adicionalmente, 10 mM de Tetrodotoxina (TTX) (Alomone, Israel) foi utilizado para confirmar que o fenótipo observado era de fato decorrente da I_NaT_.

### Análise estatística

Todos os resultados foram expressos como média ± erro padrão da média. Diferenças significativas foram determinadas usando o teste de T não pareado de duas amostras ou ANOVA de uma via para medidas repetidas, seguidos de análises post-hoc (Teste de Tukey). Valores de p < 0,05 foram considerados significativos. Os cardiomiócitos de pelo menos dois corações diferentes foram usados em cada experimento.

## Resultados e Discussão

**Figura 1:**
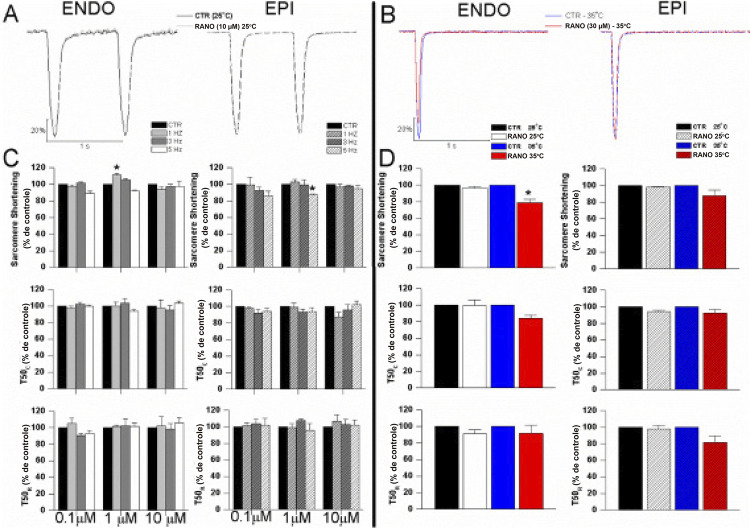
– Efeito Inotrópico da ranolazina (RANO) sobre o encurtamento do sarcômero dos cardiomiócitos do ENDO e EPI. Registros característicos de encurtamennto do sarcômero antes (preto (25ºC) e azul (35ºC)) e após (cinza claro (25ºC) e vermelho (35ºC)) exposição do cardiomiócito do ENDO (esquerda) e EPI (direita) à RANO ((A) 10 and (B) 30 µM). Efeito inotrópico da RANO (0,1, 1, e 10 µM) (C) e 30 µM (D) sobre o encurtamento do sarcômero (barras superiores); tempo normalizado de contração do sarcômero para 50% (T50C) (barras do meio) e; tempo normalizado de relaxamento do sarcômero para 50% (T50R) (barras inferiores). As barras tracejadas representam as células do EPI (n = 3–6 células/concentração). *p < 0,05, na comparação antes e depois de exposição à RANO.

Estudos anteriores demonstraram que os cardiomiócitos saudáveis apresentam I_NaT_.^6^ Além disso, um gradiente de corrente de sódio foi registrado na parede ventricular esquerda que, conforme vem sendo relatado, é maior nas células do ENDO no que nas células do EPI.^7^ Desse modo, levantamos a hipótese de que as células do ENDO apresentam maior I_NaT_ quando comparadas com as células do EPI. Uma vez que a I_NaT_ modula o [Ca^2^]i nos cardiomiócitos,^8^ a RANO seria capaz de minimizar a contração em ambos os grupos celulares, porém com maior potência nas células do ENDO e EPI. Para testar essas hipóteses, as células foram perfundidas a 25^o^C com RANO; Entretanto, a RANO não foi capaz de minimizar o encurtamento do sarcômero nos cardiomiócitos do ENDO e do EPI ( Figuras 1A e C ). Uma tendência semelhante foi observada quando os cardiomiócitos foram expostos à RANO (30 µM) e estimulados a 0,2 Hz. Quando as células do ENDO e EPI foram expostas à RANO (30 µM) e estimuladas a 0,2 Hz, usando uma solução de perfusão a 35^o^C, o encurtamento do sarcômero foi reduzido nas células do ENDO em ~21% (p < 0.05), mas não nas células do EPI ( Figuras 1B e D ). Desse modo, corroborando os achados anteriores, nossos resultados sugerem que as células saudáveis do ENDO de fato apresentam maior I_NaT_ do que as células do EPI. Entretanto, também é importante observar que a RANO (30 µM) também poderia bloquear a corrente de cálcio tipo L nos cardiomiócitos.^9^

**Figura 2:**
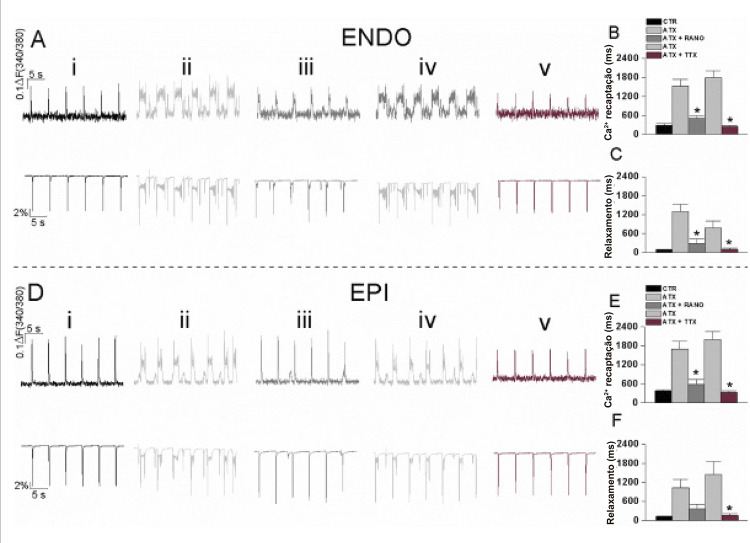
– Ação da ranolazina (RANO) nos cardiomiócitos do ENDO e EPI expostos ao ATX-II e estimulados a 0,2 Hz. Traços característicos de transientes de cálcio (traços superiores) e encurtamento dos sarcômeros dos cardiomiócitos (traços inferiores) após contato com solução de Tyrode (i), ATX-II (6 nM) (ii), ATX-II (6 nM) + RANO (30 µM) (iii), ATX-II (6 nM) (iv), e ATX-II (6 nM) + TTX (10 µM) (iv) nas células do ENDO (A) e EPI (D). Tempo para 90% de receptação de Ca^2+^ nas células do ENDO (n = 8 células) (B) e EPI (n = 6 células) (E). Tempo para 90% de relaxamento do sarcômero nas células do ENDO (C) e EPI (F). * p < 0,05, quando comparado com o grupo ATX-II.

Para melhor compreensão do mecanismo subjacente ao encurtamento do sarcômero induzido por RANO, experimentos posteriores foram realizados a 35^O^C. Os cardiomiócitos foram carregados com Fura-2/AM para monitorar a oscilação de cálcio durante a contração celular, e as células foram expostas à ATX-II para aumentar a I_NaT_ e induzir um fenótipo de SQTL tipo 33 ( Figura 2 ). As células do ENDO ( Figuras 2A, B e C ) e do EPI ( Figuras 2D, E e F ) expostas ao ATX-II mostraram evidentes perturbações do cálcio e arritmias mecânicas simultâneas. A RANO (30 µM) minimizou expressivamente o fenótipo arrítmico induzido por ATX-II em ambos os grupos celulares para extensões similares. Para confirmar que o fenótipo arrítmico observado nos nossos experimentos foram realmente atribuídos à I_NaT_, as células foram expostas ao ATX-II (6 nM) [ Figura 2A (iv) e Figura 2D (iv) ], após exposição a 10 µM de TTX e ATX-II (6 nM) [ Figura 2A (v) e Figura 2D (v) ]. Os resultados confirmaram que o fenótipo arrítmico observado foi decorrente do incremento da I_NaT_. Apesar de as células do ENDO dos ratos ter apresentado maiores correntes de sódio em relação às células do EPI,^7 , 10^ o fenótipo arrítmico induzido por ATX-II e a extensão dos efeitos antiarrítmicos da Raneza foram semelhantes em ambos os grupos celulares.

Curiosamente, o intervalo de concentração terapêutica da RANO é de 1–10 µM.^11^ A aparente discrepância no potencial da RANO pode ser explicada pelo fato de que o ATX-II nas doses de 1–10 nM induz maior I_NaT_ nos cardiomiócitos do que aquele observado nas doenças cardiovasculares.^3 , 6^

## Conclusão

A RANO exerceu pouca influência sobre o encurtamento do sarcômero de cardiomiócitos saudáveis e suprimiu as arritmias induzidas por I_NaT_ a extensões semelhantes nas células do ENDO e EPI.

## References

[1] Deo R, Albert CM (2012). Epidemiology and genetics of sudden cardiac death. Circulation.

[2] Bohnen MS, Peng G, Robey SH, Terrenoire C, Iyer V, Sampson KJ (2017). Molecular pathophysiology of congenital long QT syndrome. Physiol Rev.

[3] Clark RB, Giles WR (2016). Current-voltage relationship for late Na(+) current in adult rat ventricular myocytes. Curr Top Membr.

[4] Santos-Miranda A, Cruz JS, Roman-Campos D (2015). Electrical properties of isolated cardiomyocytes in a rat model of thiamine deficiency. Arq Bras Cardiol.

[5] Santos MS, Oliveira ED, Santos-Miranda A, Cruz JS, Gondim ANS, Menezes JER (2017). Dissection of the effects of quercetin on mouse myocardium. Basic Clin Pharmacol Toxicol.

[6] Iyer V, Roman-Campos D, Sampson KJ, Kang G, Fishman GI, Kass RS (2015). Purkinje cells as sources of arrhythmias in long QT syndrome type 3. Sci Rep.

[7] Rosati B, Grau F, McKinnon D (2006). Regional variation in mRNA transcript abundance within the ventricular wall. J Mol Cell Cardiol.

[8] Fraser H, Belardinelli L, Wang L, Light PE, McVeigh JJ, Clanachan AS (2006). Ranolazine decreases diastolic calcium accumulation caused by ATX-II or ischemia in rat hearts. J Mol Cell Cardiol.

[9] Allen TJ, Chapman RA (1996). Effects of ranolazine on L-type calcium channel currents in guinea-pig single ventricular myocytes. Br J Pharmacol.

[10] Honen BN, Saint DA (2002). Heterogeneity of the properties of INa in epicardial and endocardial cells of rat ventricle. Clin Exp Pharmacol Physiol.

[11] Chaitman BR, Skettino SL, Parker JO, Hanley P, Meluzin J, Kuch J (2004). Anti-ischemic effects and long-term survival during ranolazine monotherapy in patients with chronic severe angina. J Am Coll Cardiol.

