# Supplementary material for: Ações Transmurais Inotrópicas e Antiarrítmicas da Ranolazina em um Modelo Celular da Síndrome do QT Longo Tipo 3
Source: Arq Bras Cardiol. 2020 May 12;114(4):732–5. [Article in Portuguese] doi: 10.36660/abc.20190220 (PMC9744353; doi:10.36660/abc.20190220)
Supplement: Translation

# Translation: Inotropic and Antiarrhythmic Transmural Actions of Ranolazine in a Cellular Model of Type 3 Long QT Syndrome

**Authors:** Victor Martins Miranda, Samuel Santos Beserra, Danilo Roman Campos

**Affiliations:** 1 Brazil Universidade Federal de São Paulo, Departamento de Biofísica, Edifício de Ciências Biomédicas, São Paulo, SP - Brazil

**Keywords:** Arrhythmias, Type 3 Long QT Syndrome, ATX-II, Late Sodium current, Ranolazine, Contraction

## Abstract

Ranolazine (RANO) prevents cardiac arrhythmia by blocking the late sodium current (I_NaL_). A transmural gradient of Nav1.5 is found in the left ventricular wall of the heart. Thus, we investigated the effects of RANO in healthy cardiomyocytes and in a cellular model of type 3 long QT syndrome (LQT3). We used isolated endocardium (ENDO) and epicardium (EPI) cells and a video edge detection system and fluorescence microscopy to monitor calcium transients. RANO (0.1, 1, 10 and 30 uM, at 25^o^C) at a range of pacing frequencies showed a minor impact on both cell types, but RANO at 30uM and 35^o^C for ENDO cells attenuated sarcomere shortening by~21%. Next, to mimic LQT3, we exposed ENDO and EPI cells to anemone toxin II (ATX-II), which augments I_NaL_. Cellular arrhythmias induced by ATX-II were abrogated by RANO (30 µM) at 35^o^C. Based on our results we can conclude that RANO has a minor impact on sarcomere shortening of healthy ENDO and EPI cells and it abrogates arrhythmias induced by I_NaL_to a similar level in ENDO and EPI cells.

## Introduction

Arrhythmia in cardiovascular diseases is one of the leading causes of death worldwide.^1^ The antiarrhythmic action of RANO is attributed to reduction in the slow inactivating component of cardiac inward current through Nav1.5, known as the late sodium current (I_NaL_).^2^ Despite major advances in the understanding of molecular mechanisms underlying RANO action, whether RANO exhibits a transmural action in heart muscle cells remains uncertain. Therefore, in the present study our hypothesis is that RANO has transmural action on healthy field-stimulated endocardium (ENDO) and epicardium (EPI) cells and also on arrhythmias and calcium disturbance induced by anemone toxin II (ATX-II),^3^ which increases I_NaL_and mimics several aspects of type 3 long QT syndrome (LQT3), a diseased linked to increased I_NaL_in heart cells.^2^

## Methods

### Animals

Male Wistar rats (160–250 g; 5–7-week old) were used in the experiments. All experimental procedures were performed in accordance with institutional guidelines, and the study was approved by the local ethical review committee. Cardiomyocytes were isolated as previously described.^4^

### Sarcomere shortening and calcium transient

Experiments were conducted as previously described by our group.^5^ Cells were perfused with RANO (Alomone, Israel) at 0.1, 1, 10, or 30 µM from a 10 mM stock solution. Data were normalized as the function of sarcomere contraction before RANO exposure. To access the antiarrhythmic effect of RANO following exposure to 6 nM ATX-II (Alomone, Israel), the times to 90% sarcomere relaxation (T90R) and calcium reuptake (T90Ca^2^) were recorded as arrhythmic indexes. In addition, 10 mM tetrodotoxin (TTX) (Alomone, Israel) was used to confirm that the observed phenotype was indeed due to I_NaL_.

### Statistical analysis

All results are expressed as mean ± standard error of the mean. Significant differences were determined using two-sample t-test or one-way ANOVA with repeated measures, followed by Tukey’s post hoc test. P < 0.05 was considered significant. Cardiomyocytes from at least two distinct hearts were used in each experiment.

## Results and discussion

Previous studies have shown that healthy cardiomyocytes exhibit I_NaL_.^6^ Moreover, a gradient of sodium current has been recorded in the left ventricular wall, and it has been reported to be larger in ENDO cells than in EPI cells.^7^ Thus, we hypothesized that ENDO cells present larger I_NaL_than EPI cells. Since I_NaL_modulates [Ca^2^]i in cardiomyocytes,^8^ RANO would be able to attenuate contraction in both cell groups, although with greater potency in ENDO cells than in EPI cells. To test this hypothesis, cells were perfused at 25^o^C with RANO; however, RANO could not attenuate sarcomere shortening in ENDO and EPI cardiomyocytes ( Figures 1 A and C ). A similar trend was observed when cardiomyocytes were exposed to 30 µM RANO and paced at 0.2 Hz. When ENDO and EPI cells were exposed to 30 µM RANO and paced at 0.2 Hz using a superfusion solution at 35^o^C, cell shortening was attenuated in ENDO cells by ~21% (p < 0.05) but not in EPI cells ( Figures 1B and D ). Thus, corroborating the previous findings, our results suggest that healthy ENDO cells indeed present larger I_NaL_than EPI cells. However, it is also important to note that 30 µM RANO could also block L-type calcium current in cardiomyocytes.^9^

**Figure 1:** – Inotropic effect of ranolazine (RANO) on sarcomere shortening of ENDO and EPI cardiomyocytes. Representative sarcomere shortening recordings before (black (25ºC) and blue (35ºC)) and after (light gray (25ºC) and red (35ºC)) exposure of ENDO (left) and EPI (right) cardiomyocyte to RANO ((A) 10 and (B) 30 µM). Inotropic effect of 0.1, 1, and 10 µM RANO (C) and 30 µM (D) on sarcomere shortening (upper bars); Normalized time to 50% sarcomere contraction (T50C) (middle bars)) and; normalized time to 50% of sarcomere relaxation (T50R) (bottom bars) Hatched bars represent EPI cells (n = 3–6 cells/concentration). *p < 0.05 comparing before and after RANO exposure.

To better understand the mechanism underlying sarcomere shortening induced by RANO, subsequent experiments were performed at 35^o^C. Cardiomyocytes were loaded with Fura 2-AM to monitor calcium oscillation during cell contraction, and cells were exposed to ATX-II to increase I_NaL_and induce an LQT3 phenotype3 ( Figure 2 ). ENDO ( Figures 2A, B and C ) and EPI ( Figures 2D, E and F ) cells exposed to ATX-II showed clear calcium disturbances and simultaneous mechanical arrhythmias. RANO (30 µM) strongly attenuated the arrhythmic phenotype induced by ATX-II in both cell groups to a similar extent. To confirm that the arrhythmic phenotype observed in our experiments was truly attributed to I_NaL_, cells were exposed to 6 nM ATX-II [ Figure 2A (iv) and Figure 2D (iv) ], following exposure to 10 µM TTX and 6 nM ATX-II [ Figure 2A (v) and Fig 2D (v) ]. The results confirmed that the observed arrhythmic phenotype occurred due to I_NaL_augmentation. Despite the fact that rat ENDO cells present larger sodium currents than EPI cells,^7 , 10^ the arrhythmic phenotype induced by ATX-II and the extent of antiarrhythmic effects of RANO were similar in both cell groups.

**Figure 2:** – Action of ranolazine (RANO) in ENDO and EPI cardiomyocytes exposed to ATX-II and paced at 0.2 Hz. Representative traces of calcium transients (upper traces) and cardiomyocyte sarcomere shortening (lower traces) following exposure to Tyrode’s solution (i), 6 nM ATX-II (ii), 6 nM ATX-II + 30 µM RANO (iii), 6 nM ATX-II (iv), and 6 nM ATX-II + 10 µM TTX (iv) in ENDO (A) and EPI (D) cells. Time to 90% of Ca2+ reuptake in ENDO (n = 8 cells) (B) and EPI (n = 6 cells) (E). Time to 90% sarcomere relaxation in ENDO (C) and EPI (F) cells. * p < 0.05 compared to the ATX-II group.

Interestingly, the therapeutic concentration range of RANO is 1–10 µM.^11^ The apparent discrepancy in RANO potency may be explained by the fact that ATX-II at doses of 1–10 nM induces larger I_NaL_in cardiomyocytes than that observed in cardiovascular disease.^3 , 6^

## Conclusion

RANO exerted a minor impact on sarcomere shortening of healthy cardiomyocytes and abrogated arrhythmias induced by I_NaL_to a similar extent in ENDO and EPI cells.
